# An improved parallel fuzzy connected image segmentation method based on CUDA

**DOI:** 10.1186/s12938-016-0165-2

**Published:** 2016-05-12

**Authors:** Liansheng Wang, Dong Li, Shaohui Huang

**Affiliations:** Department of Computer Science, School of Information Science and Engineering, Xiamen University, Xiamen, China

**Keywords:** Fuzzy connectedness, CUDA, Vessel segmentation

## Abstract

**Purpose:**

Fuzzy connectedness method (FC) is an effective method for extracting fuzzy objects from medical images. However, when FC is applied to large medical image datasets, its running time will be greatly expensive. Therefore, a parallel CUDA version of FC (CUDA-kFOE) was proposed by Ying et al. to accelerate the original FC. Unfortunately, CUDA-kFOE does not consider the edges between GPU blocks, which causes miscalculation of edge points. In this paper, an improved algorithm is proposed by adding a correction step on the edge points. The improved algorithm can greatly enhance the calculation accuracy.

**Methods:**

In the improved method, an iterative manner is applied. In the first iteration, the affinity computation strategy is changed and a look up table is employed for memory reduction. In the second iteration, the error voxels because of asynchronism are updated again.

**Results:**

Three different CT sequences of hepatic vascular with different sizes were used in the experiments with three different seeds. NVIDIA Tesla C2075 is used to evaluate our improved method over these three data sets. Experimental results show that the improved algorithm can achieve a faster segmentation compared to the CPU version and higher accuracy than CUDA-kFOE.

**Conclusions:**

The calculation results were consistent with the CPU version, which demonstrates that it corrects the edge point calculation error of the original CUDA-kFOE. The proposed method has a comparable time cost and has less errors compared to the original CUDA-kFOE as demonstrated in the experimental results. In the future, we will focus on automatic acquisition method and automatic processing.

## Background

Vessel segmentation is important for evaluation of vascular-related diseases and has applications in surgical planning. Vascular structure is a reliable mark to localize a tumor, especially in liver surgery. Therefore, accurately extracting the liver vessel from CT slices in real time is the most important factor in preliminary examination and hepatic surgical planning.

In recent years, many methods of vascular segmentation have been proposed. For example, Gooya et al. [1] proposed a level-set based geometric regularization method for vascular segmentation. Yi et al. [2] used a locally adaptive region growing algorithm to segment vessel. Jiang et al. [3] employed a region growing method based on spectrum information to perform vessel segmentation.

In 1996, Udupa et al. [4] addressed a theory of fuzzy objects for n-dimensional digital spaces based on a notion of fuzzy connectedness of image elements and presented algorithms for extracting a specified fuzzy object and identifying all fuzzy objects present in the image data. Lots of medical applications of the fuzzy connectedness are proposed, including multiple abdominal organ segmentation [5], tumor segmentation [6], vascular segmentation in liver, and so on. Based on fuzzy connectedness algorithm, Harati et al. [6] developed a fully automatic and accurate method for tumor region detection and segmentation in brain MR images. Liu et al. [7] presented a method for brain tumor volume estimation via MR imaging and fuzzy connectedness.

However, with the size of medical data increasing, the sequential FC algorithm, which depends on the sequential performance of CPU, is greatly time-consuming. On the other hand, parallel technology developments in many domains, such as high-through DNA sequence alignment using GPUs [8], accelerating advanced MRI reconstructions on GPUs [9]. Therefore, some researchers proposed parallel implementations of FC. An OpenMP-based FC was proposed in 2008, the authors adapted a sequential fuzzy segmentation algorithm to multiprocessor machines [10]. Thereafter, Zhuge et al. [11] addressed a CUDA-kFOE algorithm which is based on NVIDIA’s compute unified device architecture (CUDA) platform. CUDA-kFOE computes the fuzzy affinity relations and the fuzzy connectedness relations as CUDA kernels and executes them on GPU. The authors improved their method in 2011 [12] and 2013 [13]. However, their methods has expensive computational cost because their method is in an iterative manner and lacks of interblock communication on the GPU [13].

In this paper, we proposed a novel solution to the limited communication capability between threads of different blocks. The purpose of our study is to improve the implementation of CUDA-kFOE and enhance the calculation accuracy on GPU by CUDA. The main contributions of the proposed method are in two folds. Firstly, the improved method doesn’t need large memory for large data set since we use a look up table. Secondly, the error voxels because of asynchronism are updated again and corrected in the last iteration of the proposed method.

The paper is organized as follows. In "Background" section, we first summarize the literature of fuzzy connectedness and the CPU-based FC algorithms. Then a brief description of fuzzy connectedness and the original CUDA-kFOE is presented in the "Fuzzy connectedness and CUDA executing model" and "Previous work" sections respectively. The proposed improved CUDA-kFOE is explained in the "Methods" section. The experiments and conclusion are given in the "Results and discussion" and "Conclusion" sections respectively.

## Fuzzy connectedness and CUDA executing model

### Fuzzy connectedness

Fuzzy connectedness segmentation method [14] was first proposed by Udupa et al. in 1996. The idea of the algorithm is by comparing connectivity of seed points between target area and background area to separate the target and background.

Let’s define *X* be any reference set. Fuzzy subset *A* of *X* is a set of ordered pairs,

1 $$\begin{aligned} A=\left\{ x,\mu _{A}(x)|x\in X\right\} \end{aligned}$$

where $\mu _{A}:X\rightarrow [0,1]$ is the member function of *A* in *X*. A fuzzy relation $\rho$ in *X* is a fuzzy subset of $X\times X$, $\rho =\left\{ \left( x,y \right) ,\mu _{\rho }\left( x,y \right) |x,y\in X \right\}$, where $\mu _\rho :X\times X\rightarrow [0,1]$.

In addition, $\rho$ is reflexive if $\forall x, \forall x\in X, \mu _\rho \left( x,x \right) =1$; $\rho$ is symmetric, if $\forall x,y\in X, \mu _\rho \left( x,y \right) =\mu _\rho \left( y,x \right)$; $\rho$ is transitive, if $\forall x,z \in X, \mu _\rho \left( x,z \right) =max _{y \in x}[min(\mu _\rho \left( x,y \right) ,\mu _\rho (y,z))]$.

Let $C=(C,f)$ be a scene of $(Z^n,a)$, and if any fuzzy relation *k* in *C* is reflexive and symmetric, we said *k* to be a fuzzy spel affinity in *C*. We define $\mu _k$ as

2 $$\begin{aligned} \mu _k(c,d)=\mu _\alpha (c,d)\sqrt{g_{1}(f(c),f(d))g_{2}(f(c),f(d))} \end{aligned}$$

where $g_1,g_2$ are Gaussian function represented by $\frac{f(c)+f(d)}{2}$ and $\frac{|f(c)-f(d)|}{2}$ respectively. The mean and variance of $g_1$ are computed by the intensity of objects surrounded in fuzzy scene, $g_2$ is a zero-mean Gaussian.

### CUDA executing model

**Fig. 1:**
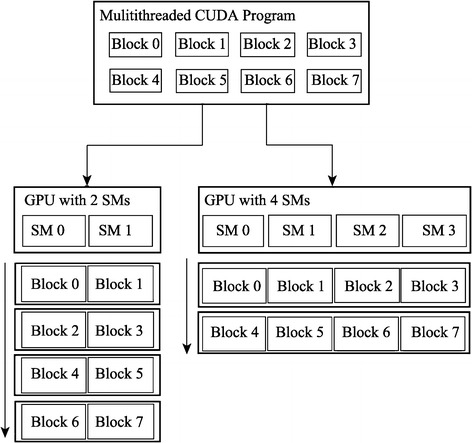
Automatic scalability in CUDA [17]

The basic strategy of CUDA is for all computing threads to run concurrently in logic. Actually, tasks will divide thread blocks according to the equipments of different CUDA devices, and GPU will automatically distribute task blocks to each stream multiprocessor (SM). Figure 1 shows a procedure of blocks divided from software level to hardware level. In this procedure, all SMs will run in parallel independently. This means any task blocks in different SMs won’t execute synchronization instructions [15].

## Previous work

In this section, a brief introduction of the CUDA-kFOE Algorithm proposed by Ying Zhuge et al. is presented, in which the kFOE is well parallelized. The CUDA-kFOE algorithm consists of two parts.

1. Affinity computation. We can use Eq. (2) to compute the affinity of voxel (*c*, *d*), and the result of affinity $\mu _k (c,d)$ is stored in the special GPU device memory.
2. Updating fuzzy connectivity. The nature of computation for the fuzzy connectivity is a single-source-shortest-path (SSSP) problem. How to parallelize the SSSP is a challenge problem. Fortunately, CUDA-based SSSP algorithm proposed by Harish and Narayanan solves the problem [16]. With the computing capability of Eq. (2), the atomic operations are employed to solve multiple threads by accessing the same address conflict which basically achieve SSSP parallelization, and the algorithm is presented in [11].

## Methods

### Performance analysis and improvement

In the first step of CUDA-kFOE algorithm, we need release enormous memory space to store the six-adjacent affinity when computing large CT series data. In addition, CUDA-kFOE will suffer from errors in some voxels in the scenario of different blocks hard to execute synchronously.

In order to overcome these drawbacks of the CUDA-kFOE algorithm, in this section, we propose an improved double iterative method which can be implemented easily and has more accurate performance. The main advantages of the improved method are as follows.

1. The proposed algorithm needs less memory compared to CUDA-kFOE when processing large data sets. (We change the affinity computation strategy by using look up table for memory reduction).
2. The proposed algorithm doesn’t need CPU involved to handle extra computing and therefore achieve more accurate results. (The main idea is to process twice the error voxels because of asynchronism. Therefore those error voxels will be processed again in the last iteration).

**Fig. 2:**
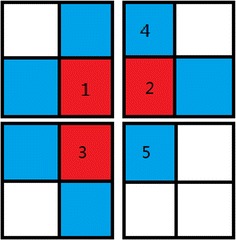
Illustration of edge points processing situation.* Red points* means their neighborhood points are needed to be updated.* Blue points* means they are being updated

Let’s analyze the performance of CUDA-kFOE. Considering a single seed to start the CUDA-kFOE algorithm, and using breadth-first for computing fuzzy scenes. Figure 2 illustrates the processing of edge points, where red points represent its neighbors required to be updated and blue points represent being updated points. If the red points denote fuzzy affinity for propagation outside, the competition problem will be triggered when red points reach the blocks’ edge. The reason is that the fuzzy affinity must be propagated between different blocks. Since the procedure of outward propagation of seed point looks like a tree shape and therefore the path will not appear in a circle. Thus the calculation procedure can be seen as the generation of tree structure which is built on seed points as the tree root.

In Fig. 2, pixel 1, (2, 4), 3 and 5 locate at different thread blocks. Pixel 1, 2 and 3 are in $C_1$(c) array and pixel 4 and 5 are updated points which are the neighbors of pixel 2. Considering the worst situation: because the runnings of thread blocks are disorder, when judging $f_{min}>f(e)$, pixel 5 will be influenced by pixel 2 and 3 together. The running orders have six situations:

- $$\, 2\rightarrow 5, 3\rightarrow 5;$$
- $$\, 3\rightarrow 5, 2\rightarrow 5;$$
- $$\, 1\rightarrow 3, 1\rightarrow 2, 3\rightarrow 5, 2\rightarrow 5;$$
- $$\, 1\rightarrow 3, 1\rightarrow 2, 2\rightarrow 5, 3\rightarrow 5;$$
- $$\, 2\rightarrow 1, 2\rightarrow 5, 1\rightarrow 3, 3\rightarrow 5;$$
- $$\, 3\rightarrow 1, 3\rightarrow 5, 1\rightarrow 2, 2\rightarrow 5;$$

Because updating the pixel 5 only need selecting the max values of fuzzy affinity between pixel 1 and 2, the orders of situation (a) and (b) won’t influence the propagating result of fuzzy affinity. Therefore, situation (a) and (b) won’t generate errors because of thread block asynchrony. In the situation (c) and (d), if the pixel 1 doesn’t influence the values of pixel 2 and 3, the results are the same as the situation (a) and (b). However, If pixel 1 influences the pixel 2 or 3, the pixel 5 will be influenced by updating the pixel 2 and 3. At this condition, if run $2\rightarrow 5$, $3\rightarrow 5$ or $3\rightarrow 5$, $2\rightarrow 5$ first, new value of pixel won’t reach pixel 5, thus pixel 5 can’t compute the correct value. Therefore, we can run a correction iterator to propagate the correct value of pixel 1. Double iterations can solve the problem of situation (c) and (d). In the situation (e) and (f), pixels will cross 3 thread blocks. It’s the same situation as (c) and (d), thus we can run triple iterations to solve the asynchronous problem.

### Improved algorithm and implementation

**Fig. 3:**
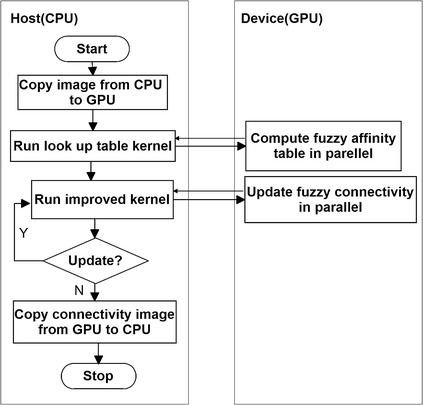
The flow char of improved CUDA-kFOE

The flow chart of improved GPU implementation is illustrated in Fig. 3, which is modified from Ref. [13]. The pseudo code of the proposed method is given in the following algorithm.

As shown in the procedure of the algorithm, improved CUDA-FOE is an iteration algorithm. In the first iteration, only one voxel will participate in computing affinity and updating the six-adjacent connectivity. While the number of iteration increase, more and more voxels will be computed in parallel until there is no any update operation from all threads, which means every voxel value in $C_1$ is all false. In the step 6 of algorithm improved CUDA-kFOE, we use atomic operation for consistency [16] since more than one thread in update operation may access the same address simultaneously. In addition, the edges of different blocks can not be easily controlled which may cause error values for the voxels at the edge of blocks. Therefore we use two iterations to solve the problem.

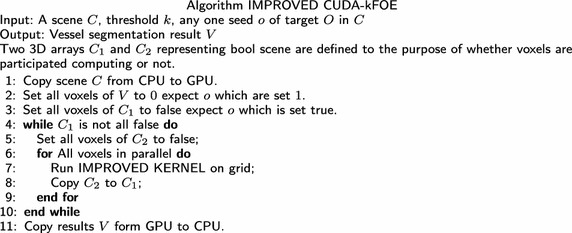

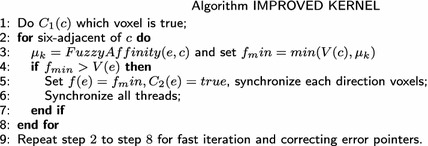





## Results and discussion

In the experiments, the accuracy of the proposed method is evaluated by compared to original CUDA-kFOE and the CPU version of FC at the same condition. The CPU version source code of fuzzy connectedness is from Insight Segmentation and Registration Toolkit (ITK).

The experiments use a computer of DELL Precision WorkStation T7500 Tower which is equipped with two quad-cores 2.93 GHz Intel Xeon X5674 CPU. It runs Windows 7 (64 bit) with 48 GB device memory. We use NVIDIA Quadro 2000 for display and NVIDIA Tesla C2075 for computing. The NVIDIA Tesla C2075 is equipped with 6 GB memory and 14 multiprocessors, in which each multiprocessor consists of 32 CUDA cores. Table 1 shows the data set used in the experiments and the results of CPU version, original GPU version and improved GPU version in running time and accuracy. Error pointers is defined as the difference between CPU version and GPU version and its result is displayed in a new image.

**Table 1:** Experimental data set and performance comparison of original and improved CUDA-kFOE

Dataset	Small	Medium	Large
Seed position	(166, 224, 88)	(189, 245, 175)	(220, 217, 497)
Scene domain	512 * 512 * 131	512 * 512 * 261	512 * 512 * 576
Voxel size (mm^3^)	0.69 * 0.69 * 1.0	0.70 * 0.70 * 1.0	0.87 * 0.87 * 0.8
CPU time (s)	386	783	1157
Origin GPU version (s)	6.5	15.5	39.9
Error points (original)	1169	4800	736
Improved GPU time (s)	7.2	16.8	41.9
Error points (improved)	0	1	0

**Fig. 4:**
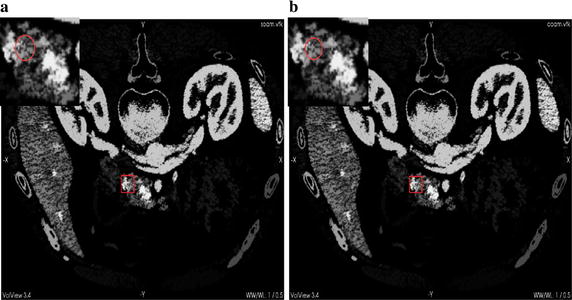
**a** The result of original CUDA-kFOE,** b** the result of improved CUDA-kFOE

Figure 4a shows the result of original CUDA-kFOE in one slice and (b) is the result of improved CUDA-kFOE. There are error points in the result of original CUDA-kFOE compared to our improved one. we choose one region with red rectangle in the results to demonstrate the error points. The region are blown up at the left-upper corner of the results, in which we can clear see there are missing pixels in the result of original CUDA-kFOE compared to the improved one.

**Fig. 5:**
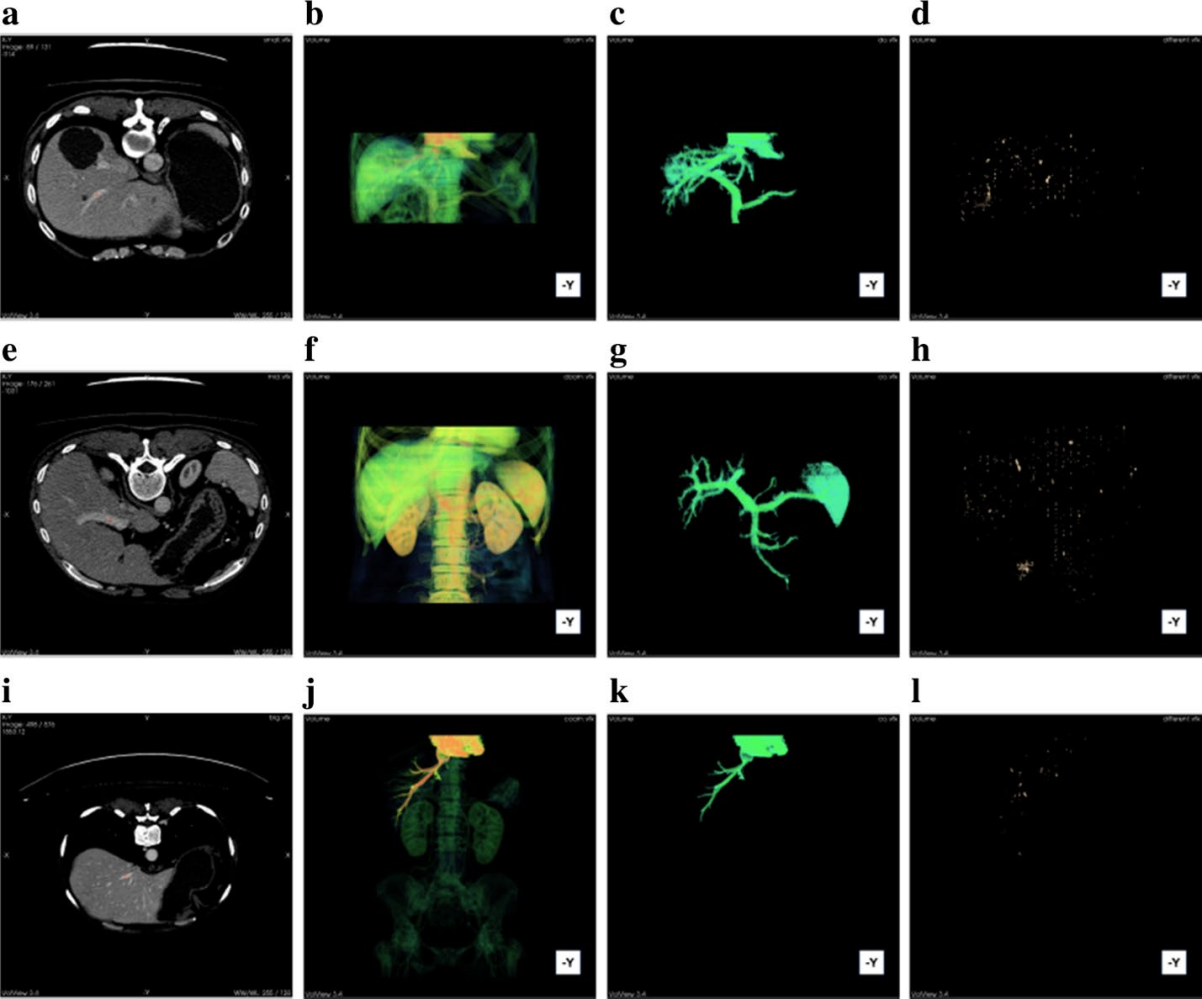
**a** One slice of origin CT series;** b** original fuzzy scenes;** c** threshold segmentation result;** d** different pointers. Images in *column* **a **are in cross sectional view. *Columns* **b, c,** and** d** are in longitudinal view of -Y direction.

Figure 5 demonstrates the performance comparison of the original CUDA-kFOE and the improved one in different size of data set. In each row, column (a) shows one slice of origin CT series; column (b) and (c) show original fuzzy scenes and threshold segmentation result respectively; column (d) is the different points of origin GPU version and CPU version. From top to bottom, the data set size is $512*512*131$ in the first row, $512*512*261$ in the second row, $512*512*576$ in the third row. It is demonstrated that the bigger vascular, the more different points generated.

**Fig. 6:**
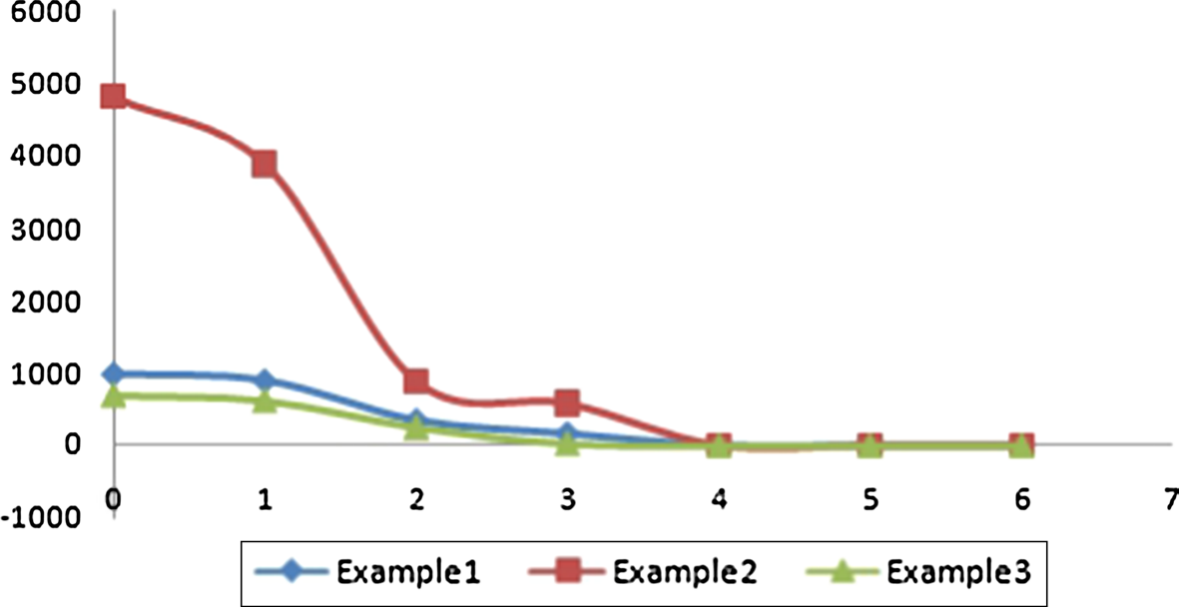
Error points of the improved method in different iteration directions

In addition, the improved method is further evaluated in different iteration directions as shown in Table 2. The results are also visualized in the Fig. 6. It is illustrated that the results have higher accuracy and less number of error points when choosing more adjacent edges during iterations.

**Table 2:** Error points of the improved method in different iteration directions

Direction	0	1	2	3	4	5	6
Small	1187	897	348	164	2	0	0
Medium	4800	3868	880	578	1	0	0
Large	693	619	254	30	0	0	0

**Fig. 7:**
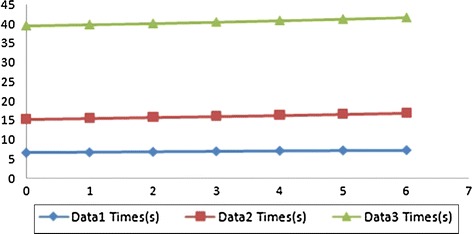
Time consuming (*Data 1* small, *Data 2* medium, *Data 3* large)

The time cost of each iteration direction is shown in the Fig. 7. For each data set, time cost slightly change while increase the iteration directions, because in the proposed twice-iteration method, most pointers reach their right values and only a few threads will participate in re-computing step.

## Conclusions

In this study, we proposed an improved CUDA-kFOE to overcome the drawbacks of the original one. The improved CUDA-kFOE is in an two iterations manner. Two advantages are in the improved CUDA-kFOE. Firstly, the improved method doesn’t need large memory for large data set since we use a look up table. Secondly, the error voxels because of asynchronism are updated again in the last iteration of the improved CUDA-kFOE. To evaluate the proposed method, three data sets of different size are used. The improved CUDA-kFOE has a comparable time cost and has less errors compared with the original one as demonstrated in the experiments. In the future, we will study automatic acquisition method and complete automatic processing.

## Abbrevations

CUDA: compute unified device architecture
FC: fuzzy connectedness
CUDA-kFOE: CUDA version of FC
CT: computed tomography
MR: magnetic resonance
SM: stream multiprocessor
